# A framework to build similarity-based cohorts for personalized treatment advice – a standardized, but flexible workflow with the R package SimBaCo

**DOI:** 10.1371/journal.pone.0233686

**Published:** 2020-05-29

**Authors:** Lucas Wirbka, Walter E. Haefeli, Andreas D. Meid

**Affiliations:** Department of Clinical Pharmacology and Pharmacoepidemiology, Heidelberg University Hospital, Heidelberg, Germany; University of California Berkeley, UNITED STATES

## Abstract

Along with increasing amounts of big data sources and increasing computer performance, real-world evidence from such sources likewise gains in importance. While this mostly applies to population averaged results from analyses based on the all available data, it is also possible to conduct so-called personalized analyses based on a data subset whose observations resemble a particular patient for whom a decision is to be made. Claims data from statutory health insurance companies could provide necessary information for such personalized analyses. To derive treatment recommendations from them for a particular patient in everyday care, an automated, reproducible and efficiently programmed workflow would be required. We introduce the R-package SimBaCo (Similarity-Based Cohort generation) offering a simple, but modular, and intuitive framework for this task. With the six built-in R-functions, this framework allows the user to create similarity cohorts tailored to the characteristics of particular patients. An exemplary workflow illustrates the distinct steps beginning with an initial cohort selection according to inclusion and exclusion criteria. A plotting function facilitates investigating a particular patient’s characteristics relative to their distribution in a reference cohort, for example the initial cohort or the precision cohort after the data has been trimmed in accordance with chosen variables for similarity finding. Such precision cohorts allow any form of personalized analysis, for example personalized analyses of comparative effectiveness or customized prediction models developed from precision cohorts. In our exemplary workflow, we provide such a treatment comparison whereupon a treatment decision for a particular patient could be made. This is only one field of application where personalized results can directly support the process of clinical reasoning by leveraging information from individual patient data. With this modular package at hand, personalized studies can efficiently weight benefits and risks of treatment options of particular patients.

## 1 Introduction

Analyses of large, routinely collected data sources can support decision-making in new patients whose data in turn contribute to that data source again [1]. Comparative effectiveness research and personalized prediction of outcomes are two major analytical applications evaluating big healthcare data repositories such as statutory health insurance databases [2]. Such information could guide medical treatment recommendations in situations with limited evidence from randomized controlled trials as frequently encountered in frailty, multimorbidity, older patients, and also children [3]. The decisive information on individual benefits and harms is similarly difficult to obtain from controlled observational studies because averaged responses in heterogeneous treatment groups might not apply to a particular patient, even if they were derived by appropriate methods approximating causal inference [4]. Reasoning based on average responses of heterogeneous populations thus tends to over-generalize effects in individual patients [5, 6]. As an intuitive solution, the data source can be personalized in accordance with individual patient characteristics to derive more specific subsets called precision cohorts. Analyses based on such precision cohorts hold the promise to improve predictions compared to models developed from an unselected source [7]. In order to achieve adequate (internal and also external) validity and transportability, it is fundamental to generate a precision cohort in a reproducible and preferably automated way [8].

Prominent examples of leveraging big data sources to inform decisions have successfully been used to support treatment decisions [3, 9]. While electronic health records (EHR) are usually rich data from rather heterogeneous in-house records [10], claims data are more similar within and across healthcare systems because they are much more standardized and structured and would thus enable common workflows to derive ready-for-analysis datasets for personalized analyses in precision cohorts. Efficient programming techniques are required, though, because claims data are usually large, often containing millions of patients, and thus making any electronic processing computationally intensive. Hence, simple and clean scripts are desirable to facilitate debugging, sharing, and reproducibility [10].

To meet these requirements, we developed the R package SimBaCo (Similarity-Based Cohort generation) for generating precision cohorts within a standardized and flexible workflow to be applied to claims data. Making use of parallel processing and best practices of efficient programming [11], the package has been tested on simulated data, which are also provided on its GitHub repository [12]. While the simulated claims data closely resemble the structure of claims data from the German healthcare system, the functions can be readily adapted to any other format (e.g., usage of ICD-9 instead of ICD-10 codes). In a methodology section, we emphasize our aims introducing two exemplary patient cases, for whom a treatment decision between two options is warranted. Our modular framework will be applied to theses cases and treatment recommendation will be derived from simulated example data. Our workflow starts with initial cohort selection and generation of corresponding data subsets. Such cohorts can be further modified (e.g., by calculation of propensity scores) according to individual user needs before they are eventually used to identify patients resembling a particular patient. Before creating similarity cohorts, we show how similarity in respect to a reference population can be visualized. We then describe the similarity search with its distinct parameters and functionalities to trim the precision cohort in the trade-off between larger sample sizes and higher grade of similarity. After summarizing the results of our exemplary analysis, we discuss potential applications and offer recommendations for the package’s implementation into research projects.

## 2 Methods

### 2.1 Exemplary cases

We introduce two exemplary patient cases in order to illustrate how SimBaCo may improve clinical decision making. Let’s assume that both exemplary patients are female and have two pre-existing conditions. Patient 1 has a history of colorectal cancer and also suffers from high blood pressure denoted by ICD-10 codes C26.0 and I10.0, respectively. Pharmacological treatment of hypertension drugs acting on the renin-angiotensin-aldosterone system denoted by the 3-digit ATC code C09. Patient 2 suffers from a mild form of diabetes mellitus type 2 (which is currently not treated with medication, but with a change of diet) and recurrent depressive episodes denoted by ICD-10 codes E11.9 and F33.8, respectively. These comorbidities would have been identified by automated calculation of the Elixhauser Comorbidity Score [13]. The year of birth of Patient 1 is 1935, Patient 2 is born in 1948.

Both patients were newly diagnosed with atrial fibrillation and therefore require appropriate anticoagulation for stroke prophylaxis [14]. The prescriber would have to make an (informed) choice what treatment option to provide. Our exemplary workflow highlights decisive steps in the decision-making process offered by the package SimBaCo to clinicians. In particular, we use a virtual situation based on simulated data to derive personalized cohorts and thus personalized evidence. In our constructed example, we aim to choose the best particular DOAK (direct acting oral anticoagulant) for each of the exemplary patient cases to prevent systemic embolism or stroke.

### 2.2 Software, installation, and performance

The SimBaCo package was developed using the R software environment in version 3.6.0 (R Foundation for Statistical Computing, Vienna, Austria) and contains the six R functions buildcohort(), Draw_Scale_Chart(), Find_Similar(), Search_IN_Dataframe(), Search_After_Index(), and Data_Plot_Similarity(), as well as the three data sets DIAGNOSES, INSURANTS, and PRESCRIPTIONS. The exemplary analysis that we present throughout the next chapters took 42.42 seconds on our computer (HP EliteDesk 800 G2 SFF, processor: Intel(R) Core(TM) i5600 Quad-Core-processor @3.2 GHz, ram: 16GB DDR4 ram). SimBaCo was optimized by efficient programming techniques and designed to run efficiently even in large data sets (such as common claims data). Among techniques for efficient programming, parallel processing of the data was extensively applied. A detailed instruction for the installation of SimBaCo can be found in S1 Appendix.

### 2.3 Reference data

The SimBaCo package is designed to work with claims data and its typical structure. Claims data collected for administrative or billing purposes usually contain separate tables from different sources (e.g., prescription claims, diagnoses from ambulatory and stationary care, or basic demographics) [15]. Following this common structure, the SimBaCo package contains simulated claims data in separate data objects related to outpatient prescriptions, outpatient or inpatient diagnoses, and basic demographic information of 10,000 patients. For intuitive understanding, these built-in data can be considered as minimal data sets, with the PRESCRIPTIONS data frame consisting of the three variables ID (the patient unique identifier), ATC code (Anatomical Therapeutic Chemical) of the prescribed drug, and the DATE of the prescription. The DIAGNOSES data frame consists of the three variables ID, ICD code (International Statistical Classification of Diseases and Related Health Problems) of the documented diagnosis, and the DATE of diagnosis, and the INSURANTS data frame consists of the variables ID, SEX, DATEOFBIRTH (the patients’ dates of birth as calendar years), and DATEOFDEATH. Example data were generated for *i* = 10,000 virtual patients (IDs) of whom 6148 were specifically designed to illustrate the example workflow used in this paper. Beginning with the PRESCRIPTIONS data frame, the first 3233 IDs were randomly assigned 1 to 10 prescription redemptions belonging to ATC code B01AE07 (dabigatran) and IDs 3234 to 6148 were assigned between 1 and 10 prescription redemptions belonging to ATC code B01AF01 (rivaroxaban). Likewise, to IDs 1 to 6148 we assigned 1 to 10 prescriptions starting with the three-digit ATC code C07 and 1 to 20 prescriptions starting with C09. Further co-medication was randomly selected by drawing between 2 and 5 ATC codes from a real-data set of a large German statutory health insurance population (the data originates from the years 2011 to 2016) [15]. In the DIAGNOSES data frame, IDs 1 to 6148 received between 2 and 5 times the diagnosis atrial fibrillation (ICD10: I48.X). In addition, between 1 and 20 diagnoses were randomly drawn from the mentioned real-data set [15] and assigned to these patients. Likewise, diagnosis dates were also randomly drawn from this data source. In the INSURANTS data frame, sex of the patients was randomly assigned, and the date of birth of the patients was randomly set to a year between 1930 and 1950. The patients’ date of death was simulated as exponentially distributed survival times using the method of Bender and colleagues [16]. Assuming a baseline death hazard of 0.0005, the linear predictor to derive survival times was designed with main effects attributed to age, the drugs with ATC codes B01AF01, B01AE07, C07, and C09, the Elixhauser derived diagnosis groups [13] for uncomplicated hypertension, diabetes, solid tumors, and metastatic cancers, as well as interaction terms between the ATC codes B01AF01 and B01AE07 with age, diabetes, hypertension, and sex. The example data created in this way will serve as an exemplary claims data basis in the following, from which the therapy recommendation for the two example cases will be derived. The way to access the example data is described in detail in S2 Appendix.

## 3 Results of an exemplary workflow

### 3.1 Initial cohort selection

Before a suitable therapy recommendation can be derived for one of the two example cases, it is of great importance to form a suitable study cohort from the entirety of the data. Since the above question is about determining the best possible DOAK therapy for patients with atrial fibrillation at first prescription, a cohort (out of the data) with a new-user design and the index date as the first prescription date of a DOAK is the best choice to derive decision support for the exemplary cases from the data. Since the DOAK prescription is intended to serve as stroke prophylaxis in patients with atrial fibrillation, the diagnosis of atrial fibrillation is another important inclusion criterion. As usual in other data-based cohort studies, one year is used as the preliminary period of observation and 18 years as the minimum age for inclusion. For this task SimBaCo provides a fast working function to create an initial study cohort according to the users intentions and requirements. The buildcohort() function enables to choose between the common "new user design" and "prevalent user design". In addition, further inclusion and exclusion criteria can be applied, such as minimum patient age or run-in period prior to follow-up. An example on how to use this function can be found in the S3 Appendix. The possible arguments of the buildcohort() function are described in more detail in S1 Table. When applying the exemplary default criteria, buildcohort() function returns a cohort with 3513 patients with the respective start dates of inclusion (DATEIndex), the first entry in the data set (STARTDATE), and the difference between these two (see S3 Appendix). With this built-in feature, our package provides an easy-to-use, easy-to-customize, and reproducible method for creating study cohorts out of claims data.

### 3.2 Data subsets and similarity visualization

Based on this cohort selection, the subsets of the source data (example data) can be derived including only those patients selected by the buildcohort()function. This can be accomplished via the function Search_IN_Dataframe(). This function returns all rows of a source data frame for which the content from a specific variable can also be found in another data frame within a specific variable. An example of this would be the selection obtained from running the buildcohort() function. This general procedure can be applied to any suitable data frame (e.g., the Find_Similar() function described later). An example on how to use this function can be found in the S4 Appendix. Of note, further steps of data preparation can be inserted at this stage. For example, propensity scores may be added or data may be matched and trimmed by means of these propensity scores. However, treatment allocation in our exemplary data was completely at random and excludes any confounding by indication in subsequent analyses by design; therefore, these options are not addressed here any further.

**Fig 1:**
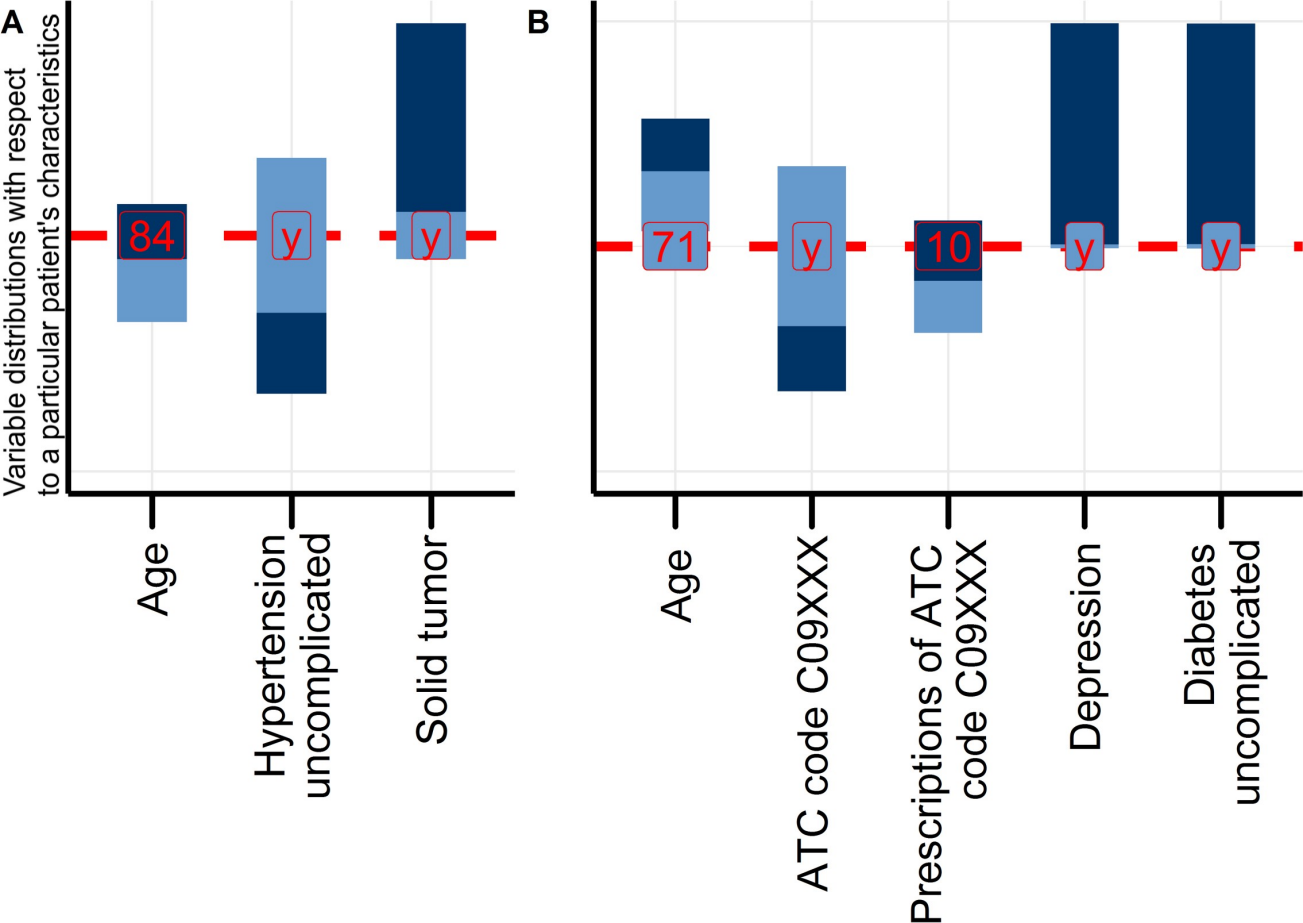
Scale charts of Patient 1 (A) and Patient 2 (B) in the initial, unselected cohort. The scale chart compares a patient’s characteristics (red vertical line) to the distribution of characteristics in the reference cohort (blue vertical bars). Each bar represents ranges derived from medians and interquartile ranges for continuous variables, and relative frequency for categorical variables. Different shades of blue indicate different categories or ranges above and below the median, respectively.

To demonstrate the typical procedure for comparing treatment options in personalized cohorts with the SimBaCo package and its example data, we introduced two exemplary patient cases for personalized analyses in two corresponding precision cohorts. The package SimBaCo provides a function to visualize the similarity between a particular case and a reference population with means, medians, and further parameters describing the distribution of variables in this sample. The reference population can be the initial study cohort obtained after running the buildcohort() function or a subsequent selection of so-called nearest patients. The Draw_Scale_Chart() function adapts the scale chart as introduced by Cahan and Cimino [17]. The function arguments are described in more detail in S2 Table. An example on how to use this function can be found in the S5 Appendix. Fig 1A presents such a scale chart corresponding to the Patient 1. Similarly, a scale chart can be drawn for example case two (Fig 1B). Both Fig 1A and 1B show selected characteristics (e.g. the age, prescribed medications or comorbidities of the exemplary patient cases etc.), of the respective cases in relation to the reference cohort. In total, neither of these two patients can be considered as a totally ‘typical’ representative of the initial cohort because their characteristics do not always match the means or most frequent values in the reference population. The aim of the next step is thus to trim the reference population according to these characteristics and thereby obtain precision cohorts that are more similar to the individual patient (considering the trade-off between higher sample sizes and higher degree of similarity), to derive individual patient-related individual therapy recommendations.

### 3.3 Generation of precision cohorts and analysis

**Fig 2:**
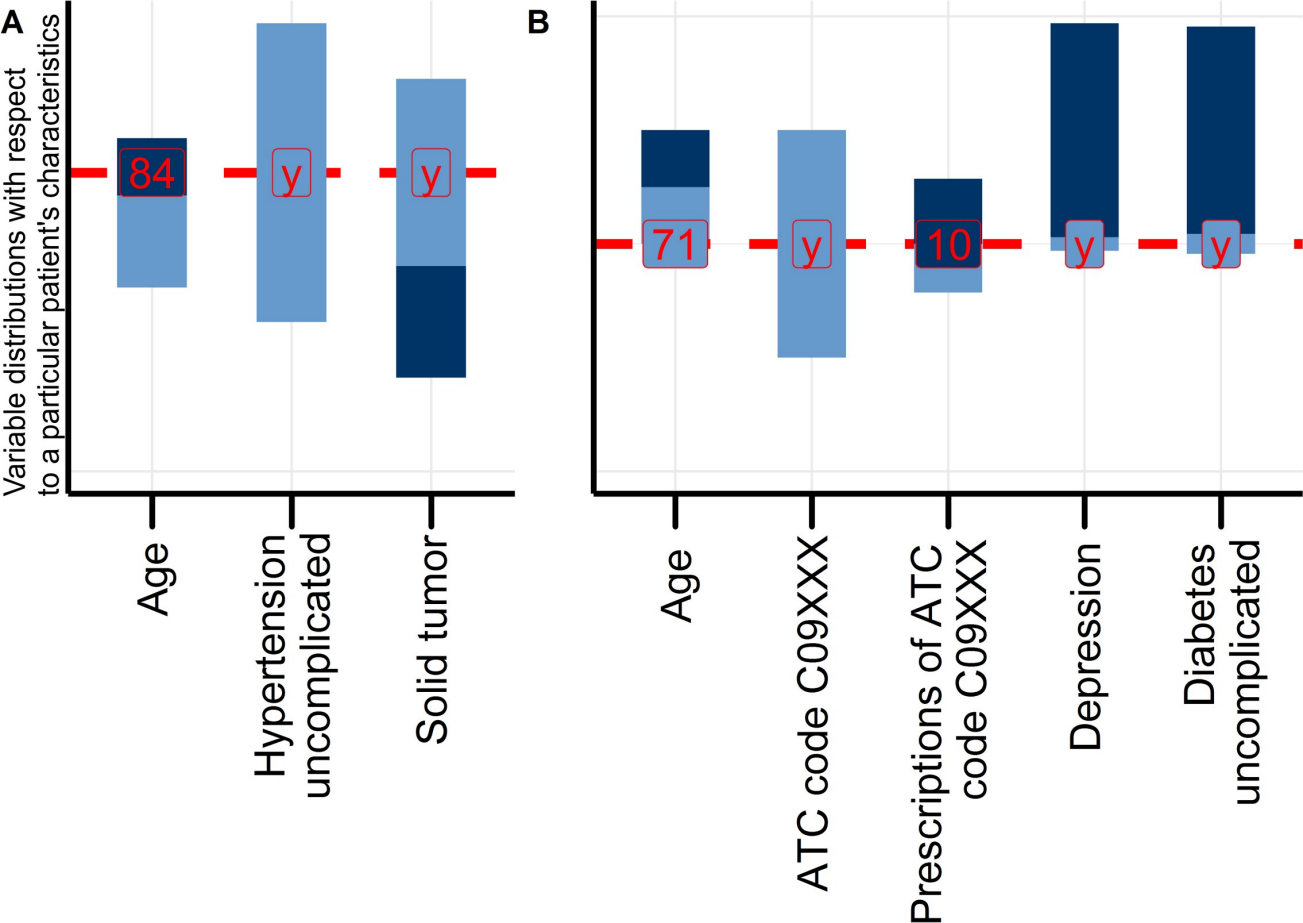
Scale charts of Patient 1 (A) and Patient 2 (B) from the respective personalized precision cohorts after the similarity search.

The scale charts indicated that the starting cohort is not yet suitable for deriving individual therapy recommendations for the exemplary patient cases. A personal treatment recommendation would require that patients in the cohort much more resemble each exemplary Patient. The SimBaCo package allows to derive suitable precision cohorts with the function Find_Similar()as a fast and reproducible solution. The function offers two types of analyses: first, the selection of closest patients as described by minimizing distance measures (e.g., the 5% of closest patients), and second, a clustering procedure using partition around medoids [18, 19] to select similar patients from the same cluster. The user can choose between two implemented distance measures, i.e., the GOWER distance and HEOM (Heterogeneous Euclidean Overlap Metric) [20, 21]. Concerning the distance-based options, this selection is guided by the fact that claims data contain differently scaled variables (i.e., categorical, continuous, and binary variables), which can be efficiently handled by these metrics. In brief, the GOWER distance uses the L1 distance (Manhattan distance) for metrically scaled variables, the Dice distance for categorically or nominally scaled variables, and then combines these two distance measures into a numerical value [20]. Concerning the cluster-based option, the K-medoids [18, 19] algorithm (in combination with the GOWER [20] distance) provides good comprehensibility and greater robustness against outliers than alternative methods, such as the K-Means. The function arguments are described in more detail in S3 Table. An example on how to use this function (to form precision cohorts) can be found in the S6 Appendix. In our example analysis, we chose the distance-based option (10% of the nearest patients) resulting in precision cohorts including 351 patients. Next, we apply the Search_IN_Dataframe() function to subdivide our preliminary data and draw again a scale chart now comparing Patient 1 and 2 with the precision cohort (Fig 2A and 2B). Comparing the scale charts before (Fig 1A and 1B) and after trimming the reference population according to similarity criteria (Fig 2A and 2B) reveals that the precision cohorts indeed more closely resemble the selected characteristics of the respective exemplary patient. Achieved similarity depends on the cut-off values in the distance measure. In our example, the ten percent nearest patients in their distance measure yielded an acceptable cut-off considering the trade-off between larger sample size and higher grade of similarity.

**Fig 3:**
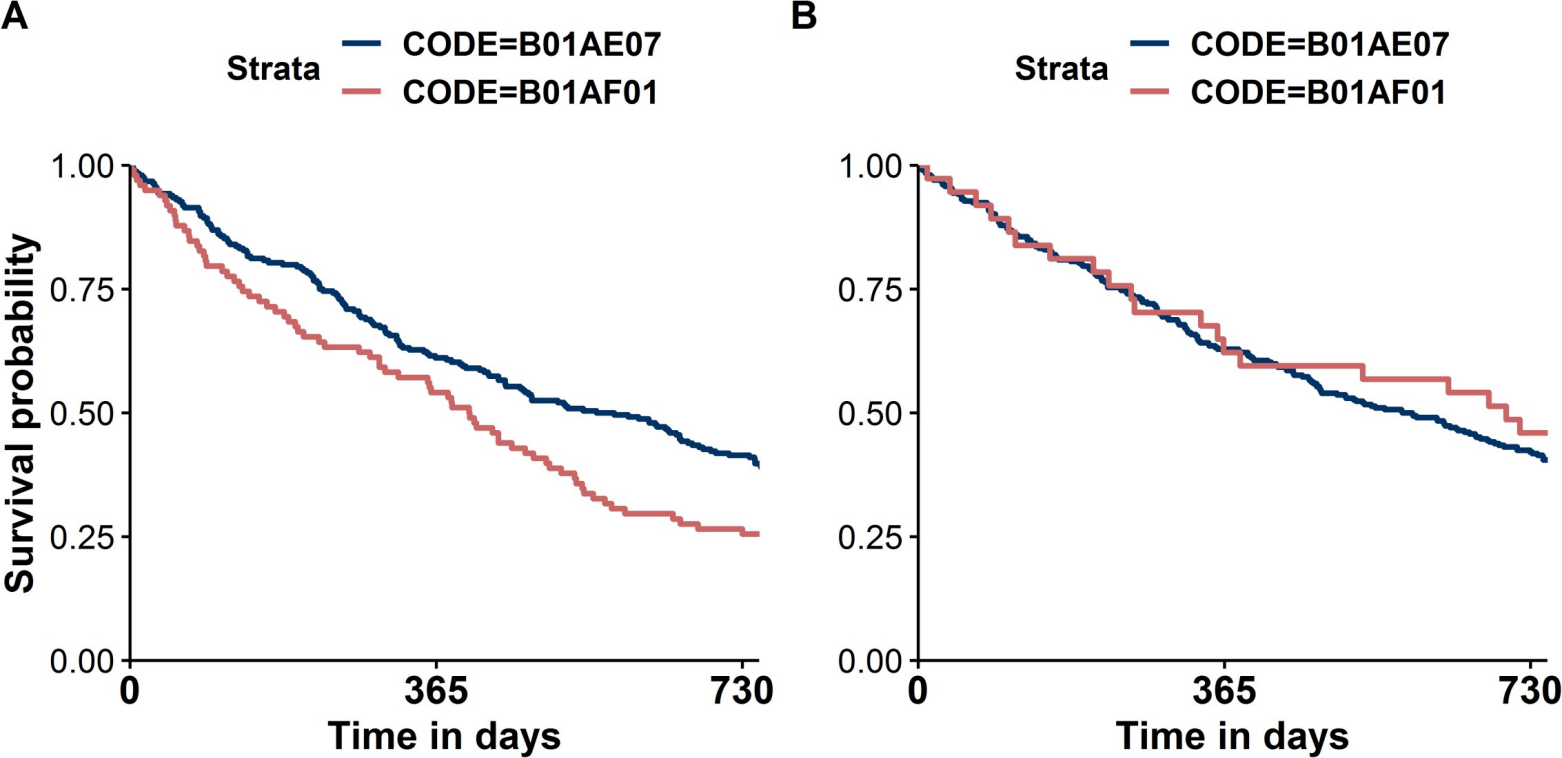
Kaplan-Meier plots comparing overall survival between two treatments (ATC codes B01AE07 and B01AF01 in personalized precision cohorts trimmed for Patient 1 (A) and Patient 2 (B)).

After successful selection and trimming of the precision cohorts, we investigate overall survival as the clinical outcome in our exemplary application of comparative effectiveness. In order to compare the overall survival in the different therapies in the precision cohorts of each case, we choose the graphical method via the Kaplan-Meier-Plot. It should be noted in addition that there are of course many other methods of deriving a therapy recommendation from the precision cohort (but these will not be discussed in detail here). Established code (Exemplary analysis code can be found in the S8 Appendix) can be applied to generate Kaplan-Meier plots (Fig 3A and 3B) but must be executed separately for each patient (Patient 1 and Patient 2).

## 4 Discussion

After completing our exemplary workflow, two remarkably different Kaplan-Meier plots resulted, indicating clearly different estimates of treatment effectiveness in dependence of the precision cohorts’ characteristics. This generic example highlights how differently clinical decisions will be made depending on the subpopulations whereupon analyses are based. Such findings could indeed support clinical decision-making and clinical reasoning. Clinical reasoning can be defined as the process by which a healthcare professional interacts with a patient when interpreting patient data, weighting the benefits and risks of treatment options, and trying to incorporate patient preferences to finally design a personalized treatment plan [22]. Prior knowledge about similar patients could certainly guide such treatment decisions for the treatment plan, as well. While the massive amounts of accumulating data far exceed our mental data processing capability and capacity, efficient electronic tools can select, sort, count, and weight data and timely provide us with the necessary information that we specifically request. SimBaCo is a prototypical example of such a tool that can support clinical reasoning in many ways, because its modular framework can be easily adjusted to various kinds of analyses. While our exemplary project addressed a simplified situation, it may serve as a basic template that can be further adjusted according to specific needs. Thus, confounding by indication as the most important aspect in comparative effectiveness research [23] could be adressed by additional interim data processing. The second major application of learning from big healthcare data is personalized prediction of outcomes [2]. Here, we are even more optimistic about potential merits of predictive performance of analyses in precision cohorts compared to unselected cohorts. While this is supported by recent proof-of-concept investigations of prognostic modeling [7], it will be interesting to test whether this will also apply to predictive modeling. It is indeed an open question if prediction models developed individually in respective precision cohorts do actually perform better in situations of treatment effect modification [24] in a real-world setting offered by claims data.

There are several reasons why we based our analysis on claims data. First of all, claims data offer a large number of patients which in turn facilitates identification of a larger number of similar patients and thus provides higher statistical power [25]. This is especially true for rare disease cases. Compared to other observational data sources, the sheer size of claims data allows for simultaneous adjustment for some confounders even in complex multivariate models [25]. Generally, data preparation and selection can follow the needs of a particular problem: Because claims data include very heterogeneous patient groups, it is likewise possible to focus on respective subgroups, such as from different areas (outpatient and inpatient data), from different facilities, or over long periods of time [25]. In addition, claims data are usually in the same structure, which makes it easier to standardize information retrieval processes [25]. However, they usually have limiations, as well. Because they are primarily collected for billing purposes (and do not contain laboratory parameters), an assessment of the severity of the disease is often not possible or only with difficulty, for example [25, 26]. Misclassification problems may arise from misdiagnoses and mis-coding [25] and information before the observation period are usually available [25, 26]. Hospital EHR, on the contrary, provide a more complete picture of the patient [26] as they can be considered as a quasi-electronic form of the patient record [27]. Those diagnosis codes are presumably more accurate and are complemented by additional data sources such as laboratory values [28]. However, this information is not standardized and therefore complicates subsequent analysis steps. With regard to the predictive accuracy of models (generated from the two data sources), though, current evidence studies suggest that the performance of EHR models is only slightly better [29].

While claims data is a very comprehensive, large, and cost-effective data source particularly suitable for the formation of precision cohorts, data availability and data privacy are central aspect to be accounted for when tools such as SimBaCo are to be implemented into routine care. Unlike the clinical trial data with explicit informed consent, the situation with claims data can be more complex in terms of data ownership, data privacy, or information identifying patients. The same applies to EHR data, with the difference that EHR data usually contains even more sensitive information. While considering the risk for misuse, adequate protection should not preclude the incredible potential of accessible data for research [30]. Overall, one could even think of a comparative evaluation of precision cohorts derived from EHR and claims data.

In conclusion, SimBaCo is a highly efficient, modular tool that enables to rapidly generate precision cohorts and apply various analysis methods to them. Derived personalized results can directly support the process of clinical reasoning because they can help interpreting individual patient data in the light of former patients by weighting benefits and risks of treatment options of this particular patient. With this modular package at hand, personalized studies of comparative effectiveness or personalized prediction models can be conducted efficiently and it will be exciting to see what benefit can be expected from this currently rarely applied technique.

## Supporting information

S1 Table buildcohort() function arguments. (DOCX)

S2 Table Draw_Scale_Chart () function arguments. (DOCX)

S3 Table Find_Similar() function arguments. (DOCX)

S4 Table Data_Plot_Similarity () function arguments. (DOCX)

S1 Appendix Procedure for the installation of SimBaCo. (DOCX)

S2 Appendix Procedure for accessing the supplied example data. (DOCX)

S3 Appendix buildcohort() function call. (DOCX)

S4 Appendix Search_IN_Dataframe() function call. (DOCX)

S5 Appendix Draw_Scale_Chart() function call. (DOCX)

S6 Appendix Find_Similar() function call. (DOCX)

S7 Appendix Draw_Scale_Chart() function call two. (DOCX)

S8 Appendix Kaplan Meier Analysis of the cohorts. (DOCX)
