# Supplementary material for: A framework to build similarity-based cohorts for personalized treatment advice – a standardized, but flexible workflow with the R package SimBaCo
Source: PLoS One. 2020 May 29;15(5):e0233686. doi: 10.1371/journal.pone.0233686 (PMC7259608; doi:10.1371/journal.pone.0233686)
Supplement: S1 Table — (DOCX) [file pone.0233686.s001.docx]

**Supplementary Table 1.** buildcohort() function arguments

| STUDYDESIGN | Studydesign, can be set to “NewUser“ or “PraevalentUser” |
| --- | --- |
| INDEXTYPE | Indextype, can be set to “ATC” or “ICD” |
| STARTDATE | STARTDATE, first date in the dataset or start date of the dataset |
| STARTDATE_Format | STARTDATE_Format, date format of the field start date |
| PRELIMINARY_TIME_SPAN | PRELIMINARY_TIME_SPAN, the minimum timespan between start date of the dataset and the index date of the patient. Also relevant for the fields EXCLUSION_ATC and EXCLUSION_ICD. |
| INDEXDATE_PRAEVALENT | INDEXDATE_PRAEVALENT, index date for all patients in the mode “PraevalentUser” |
| INDEXDATE_PRAEVALENT_FORMAT | INDEXDATE_PRAEVALENT_FORMAT, date format of the field INDEXDATE_PRAEVALENT |
| ATC_INPUT | ATC_INPUT, enter the ATC codes to filter for. Add ^ before the ATC code to search for ATC codes starting with the entered letters, e.g. “^B01” searches for all ATC codes which start with B01 |
| PRESCRIPTION | PRESCRIPTION, name of the data frame containing the prescriptions. |
| PRESCRIPTION_ATC_COLNAME | PRESCRIPTION_ATC_COLNAME, name of the column in the data frame PRESCRIPTION containing the ATC_codes. |
| PRESCRIPTION_ID_COLNAME | PRESCRIPTION_ID_COLNAME, name of the column in the data frame PRESCRIPTION containing the patient IDs |
| PRESCRIPTION_DATE_COLNAME | PRESCRIPTION_DATE_COLNAME, name of the column in the data frame PRESCRIPTION containing the PRESCRIPTION dates |
| ICD_INPUT | ICD_INPUT, enter the ICD codes to filter for. Add ^ before the ICD code to search for ICD codes starting with the entered letters , e.g. “^I48” searches for all ICD codes which start with I48 |
| DIAGNOSES | DIAGNOSES, name of the data frame containing the diagnoses. |
| DIAGNOSES_ICD_COLNAME | DIAGNOSES_ICD_COLNAME, name of the column in the data frame DIAGNOSES containing the ICD codes of the diagnoses |
| DIAGNOSES_ID_COLNAME | DIAGNOSES_ID_COLNAME, name of the column in the data frame DIAGNOSES containing the patient IDs |
| DIAGNOSES_DATE_COLNAME | DIAGNOSES_DATE_COLNAME, name of the column in the data frame DIAGNOSES containing the diagnosis dates |
| INSURANTS | INSURANTS, name of the data frame containing the patient data |
| INSURANTS_ID_COLNAME | INSURANTS_ID_COLNAME, name of the column in the data frame INSURANTS containing the patient IDs |
| INSURANTS_BIRTH_YEAR_COLNAME | INSURANTS_BIRTH_YEAR_COLNAME, name of the column in the data frame INSURANTS containing the year of birth of the patient |
| EXCLUSION_ATC | EXCLUSION_ATC, enter ATC codes which are not allowed to be prescribed in the PRELIMINARY_TIME_SPAN |
| EXCLUSION_ICD | EXCLUSION_ICD, enter ICD codes which are not allowed to be diagnosed in the PRELIMINARY_TIME_SPAN |
| MIN_INCLUSION_AGE | MIN_INCLUSION_AGE, minimum age in years to add a patient to the cohort |
| MIN_NUM_PRESCRIPTION | MIN_NUM_PRESCRIPTION, minimum number of prescriptions in each of the ATC codes to add a patient to the cohort |
