# Supplementary material for: A framework to build similarity-based cohorts for personalized treatment advice – a standardized, but flexible workflow with the R package SimBaCo
Source: PLoS One. 2020 May 29;15(5):e0233686. doi: 10.1371/journal.pone.0233686 (PMC7259608; doi:10.1371/journal.pone.0233686)
Supplement: S2 Table — (DOCX) [file pone.0233686.s002.docx]

**Supplementary Table 2.** Draw_Scale_Chart () function arguments

| SELECT_COMORBIDITY | SELECT_COMORBIDITY, could be some of the comorbidities listed in the comorbidity package. Please refer to the help section of the package comorbidity for further information. |
| --- | --- |
| PATIENT_SIMILAR_BIRTH_YEAR | PATIENT_SIMILAR_BIRTH_YEAR, the year of birth for patient to match for. |
| PATIENT_SIMILAR_SEX | PATIENT_SIMILAR_SEX, the sex of the patient to match for. |
| PATIENT_SIMILAR_INDEXDATE | PATIENT_SIMILAR_INDEXDATE, the index date of the patient to match for. |
| PATIENT_SIMILAR_INDEXDATE_FORMAT | PATIENT_SIMILAR_INDEXDATE_FORMAT, date format of the field PATIENT_SIMILAR_INDEXDATE. |
| PATIENT_SIMILAR_ATC | PATIENT_SIMILAR_ATC, the ATC codes for the patient to match for. |
| PATIENT_SIMILAR_ATC_COUNT | PATIENT_SIMILAR_ATC_MENGE, the quantity of the ATC codes in the field PATIENT_SIMILAR_ATC. |
| PATIENT_SIMILAR_ICD | PATIENT_SIMILAR_ICD, the ICD codes for the patient to match for. |
| PATIENT_DIAGNOSE_TO_DRAW | Add a diagnosis to draw in the scale chart if you didn’t like cumulated information about diagnoses in e.g. the Elixhauser criteria. |
| PRESCRIPTION | PRESCRIPTION, the name of the data frame containing the prescription data |
| PRESCRIPTION_ID_COLNAME | PRESCRIPTION_ID_COLNAME, the name of the column where the IDs in the data frame prescription. |
| PRESCRIPTION_ATC_COLNAME | PRESCRIPTION_ATC_COLNAME, the name of the column containing the ATC codes in the data frame prescription. |
| DIAGNOSES | DIAGNOSES, name of the data frame containing the diagnoses. |
| DIAGNOSES_ID_COLNAME | DIAGNOSES_ID_COLNAME, name of the column in the data frame DIAGNOSES containing the IDs |
| DIAGNOSES_ICD_COLNAME | DIAGNOSES_ICD_COLNAME, name of the column in the data frame DIAGNOSES containing the ICD codes |
| DIAGNOSES_ICD_TYPE | DIAGNOSES_ICD_TYPE, can be set to “icd10” or “icd09” |
| INSURANTS | INSURANTS, name of the data frame containing the insurants’ data |
| INSURANTS_ID_COLNAME | INSURANTS_ID_COLNAME, name of the column in the data frame INSURANTS containing the patient IDs |
| INSURANTS_BIRTH_YEAR_COLNAME | INSURANTS_BIRTH_YEAR_COLNAME, name of the column in the data frame INSURANTS containing the patients’ year of birth |
| INSURANTS_SEX_COLNAME | INSURANTS_SEX_COLNAME, name of the column in the data frame INSURANTS containing the sex of the patients |
| INSURANTS_SEX_MALE | INSURANTS_SEX_MALE, which symbol is used in INSURANTS_SEX_COLNAME to classify for male |
| INSURANTS_SEX_FEMALE | INSURANTS_SEX_FEMALE, which symbol is used in INSURANTS_SEX_COLNAME to classify for female |
| INSURANTS_INDEXDATE_COLNAME | INSURANTS_INDEXDATUM_COLNAME, name of the column in the data frame INSURANTS containing the patient index dates |
| Change_Name_Plot | Use Change_Name_Plot to change the names of the scale chart on the x-axis; format = ("a","b") |
