# Supplementary material for: A framework to build similarity-based cohorts for personalized treatment advice – a standardized, but flexible workflow with the R package SimBaCo
Source: PLoS One. 2020 May 29;15(5):e0233686. doi: 10.1371/journal.pone.0233686 (PMC7259608; doi:10.1371/journal.pone.0233686)
Supplement: S3 Table — (DOCX) [file pone.0233686.s003.docx]

**Supplementary Table 3.** Find_Similar() function arguments

| PATIENT_SELECTION | PATIENT_SELECTION, can be set to “NEW” or “IN_DF” |
| --- | --- |
| ANALYSIS_TYPE | ANALYSIS_TYPE, could be “CLUSTER” or “NEAREST”. If ANALYSIS_TYPE is set to CLUSTER and PATIENT_SELECTION is set to NEW, the entered patient is stored with the ID 999999999999 |
| DISTANCE_MEASURE | DISTANCE_MEASURE can be set to “GOWER” or “HEOM” |
| SELECT_COMORBIDITY | SELECT_COMORBIDITY can be one of the comorbidities listed in the comorbidity package. For further information see the help of the package. |
| PERCENT_CUT_OFF | PERCENT_CUT_OFF, at which percentage of the nearest patient is the cut-off |
| PATIENT_SIMILAR_BIRTH_YEAR | PATIENT_SIMILAR_BIRTH_YEAR, the year of birth for patient to match for |
| PATIENT_SIMILAR_SEX | PATIENT_SIMILAR_SEX, the sex of the patient to match for |
| PATIENT_SIMILAR_INDEXDATE | PATIENT_SIMILAR_INDEXDATE, the index date of the patient to match for |
| PATIENT_SIMILAR_INDEXDATE_FORMAT | PATIENT_SIMILAR_INDEXDATE_FORMAT, date format of the field PATIENT_SIMILAR_INDEXDATE |
| PATIENT_SIMILAR_ATC | PATIENT_SIMILAR_ATC, the ATC codes for the patient to match for |
| PATIENT_SIMILAR_ATC_COUNT | PATIENT_SIMILAR_ATC_COUNT, the quantity of the ATC codes in the field PATIENT_SIMILAR_ATC |
| PATIENT_SIMILAR_ICD | PATIENT_SIMILAR_ICD, the ICD codes for the patient to match for |
| PRESCRIPTION | PRESCRIPTION, the name of the data frame containing the prescription data |
| PRESCRIPTION_ID_COLNAME | PRESCRIPTION_ID_COLNAME, the name of the column containing the IDs in the data frame prescription |
| PRESCRIPTION_ATC_COLNAME | PRESCRIPTION_ATC_COLNAME, the name of the column containing the ATC codes in the data frame prescription |
| DIAGNOSES | DIAGNOSES, name of the data frame containing the diagnoses |
| DIAGNOSES_ID_COLNAME | DIAGNOSES_ID_COLNAME, name of the column in the data frame DIAGNOSES containing the IDs |
| DIAGNOSES_ICD_COLNAME | DIAGNOSES_ICD_COLNAME, name of the column in the data frame DIAGNOSES containing the ICD codes |
| DIAGNOSES_ICD_TYPE | DIAGNOSES_ICD_TYPE, could be set to “icd10“or “icd09” |
| INSURANTS | INSURANTS, name of the data frame containing the insurants’ data |
| INSURANTS_ID_COLNAME | INSURANTS_ID_COLNAME, name of the column in the data frame INSURANTS containing the patient IDs |
| INSURANTS_BIRTH_YEAR_COLNAME | INSURANTS_BIRTH_YEAR_COLNAME, name of the column in the data frame INSURANTS containing the patients year of birth |
| INSURANTS_SEX_COLNAME | INSURANTS_SEX_COLNAME, name of the column in the data frame INSURANTS containing the sex of the patients |
| INSURANTS_INDEXDATE_COLNAME | INSURANTS_INDEXDATUM_COLNAME, name of the column in the data frame INSURANTS containing the patient index dates |
| PATIENT_SELECTION_ID | PATIENT_SELECTION_ID, if a patient already existing in the data frame should be used enter the ID of the patient here (in combination with PATIENT_SELECTION = “IN_DF”) |
