# Supplementary material for: A framework to build similarity-based cohorts for personalized treatment advice – a standardized, but flexible workflow with the R package SimBaCo
Source: PLoS One. 2020 May 29;15(5):e0233686. doi: 10.1371/journal.pone.0233686 (PMC7259608; doi:10.1371/journal.pone.0233686)
Supplement: S4 Table — (DOCX) [file pone.0233686.s004.docx]

**Supplementary Table 4.** Data_Plot_Similarity () function arguments

| ATC_INPUT | ATC_INPUT, for the ATC codes to compare in the Kaplan-Meier plot. Add ^ before the ATC code to search for ATC codes starting with the entered letter, e.g. “^B01” searches for all ATC codes which start with B01 |
| --- | --- |
| ICD_INPUT | ICD_INPUT, the ICD code to use if OUTCOME is set to ICD or DEATH_AND_ICD |
| OUTCOME | OUTCOME, the outcome in the Kaplan-Meier-Plot. (Could be set to DEATH, ICD, DEATH_AND_ICD) |
| PRESCRIPTION | PRESCRIPTION, the name of the data frame containing the prescription data |
| PRESCRIPTION_ID_COLNAME | PRESCRIPTION_ID_COLNAME, the name of the column where the IDs are stored in the data frame prescription |
| PRESCRIPTION_CODE_COLNAME | PRESCRIPTION_CODE_COLNAME, the name of the column containing the ATC codes in the data frame prescription |
| PRESCRIPTION_CODE_DATUM_COLNAME | PRESCRIPTION_CODE_DATUM_COLNAME, the name of the column containing the prescription dates in the data frame prescription |
| PRESCRIPTION_INDEXDATUM_COLNAME | PRESCRIPTION_INDEXDATUM_COLNAME, the name of the column containing the patient index dates in the data frame prescription |
| DIAGNOSES | DIAGNOSES, name of the data frame containing the diagnoses |
| DIAGNOSES_ID_COLNAME | DIAGNOSES_ID_COLNAME, name of the column in the data frame DIAGNOSES containing the IDs |
| DIAGNOSES_CODE_COLNAME | DIAGNOSES_CODE_COLNAME, name of the column in the data frame DIAGNOSES containing the ICD codes |
| DIAGNOSES_CODE_DATUM_COLNAME | DIAGNOSES_CODE_DATUM_COLNAME, name of the column in the data frame DIAGNOSES containing the diagnoses’ dates |
| DIAGNOSES_INDEXDATUM_COLNAME | DIAGNOSES_INDEXDATUM_COLNAME, name of the data frame containing the patient index dates |
| INSURANTS | INSURANTS, name of the data frame containing the insurant data |
| INSURANTS_ID_COLNAME | INSURANTS_ID_COLNAME, name of the column in the data frame INSURANTS containing the patient IDs |
| INSURANTS_CODE_COLNAME | INSURANTS_CODE_COLNAME, name of the column in the data frame INSURANTS containing the sex of the patients |
| INSURANTS_CODE_DATUM_COLNAME | INSURANTS_CODE_DATUM_COLNAME, name of the column in the data frame INSURANTS containing the death dates |
| INSURANTS_INDEXDATUM_COLNAME | INSURANTS_INDEXDATUM_COLNAME, name of the column in the data frame INSURANTS containing the patient index dates |
