# Supplementary material for: A framework to build similarity-based cohorts for personalized treatment advice – a standardized, but flexible workflow with the R package SimBaCo
Source: PLoS One. 2020 May 29;15(5):e0233686. doi: 10.1371/journal.pone.0233686 (PMC7259608; doi:10.1371/journal.pone.0233686)
Supplement: S1 Appendix — (DOCX) [file pone.0233686.s005.docx]

**Appendix Part 1.** Procedure for the installation of SimBaCo

To install the package, we recommend the following procedure:

1. Download the SimBaCo_1.1.1.tar.gz file from our GitHub repository (https://github.com/LucasWirbka/SimBaCo.git). The .zip folder is to be downloaded via the "Clone or download" button. Then, the file SimBaCo_1.0.0.tar.gz should be moved from the .zip folder into another directory. This directory is called “path_to” in the following.
2. Make sure to have all packages listed as dependencies in our GitHub README.md.
3. Enter the following line into R: install.packages("path_to/SimBaCo_1.0.0.tar.gz", repos = NULL, type = "source")

With the command library(SimBaCo), the installed package can be loaded and is ready to use.
