# Supplementary material for: A framework to build similarity-based cohorts for personalized treatment advice – a standardized, but flexible workflow with the R package SimBaCo
Source: PLoS One. 2020 May 29;15(5):e0233686. doi: 10.1371/journal.pone.0233686 (PMC7259608; doi:10.1371/journal.pone.0233686)
Supplement: S2 Appendix — (DOCX) [file pone.0233686.s006.docx]

**Appendix Part 2.** Procedure for accessing the supplied example data

After the installation of the package, the data is accessible through the following lines of code:

PRESCRIPTION <- SimBaCo::PRESCRIPTIONS

DIAGNOSES <- SimBaCo::DIAGNOSES

VERS <- SimBaCo::INSURANTS
