# Supplementary material for: A framework to build similarity-based cohorts for personalized treatment advice – a standardized, but flexible workflow with the R package SimBaCo
Source: PLoS One. 2020 May 29;15(5):e0233686. doi: 10.1371/journal.pone.0233686 (PMC7259608; doi:10.1371/journal.pone.0233686)
Supplement: S3 Appendix — (DOCX) [file pone.0233686.s007.docx]

**Appendix Part 3.** buildcohort() function call

ICD_Entry **<-** c**(**"^I48"**)**

d **<-** buildcohort**(**STUDYDESIGN **=** "NewUser",

INDEXTYPE **=** "ICD",

STARTDATE **=** "20110101",

STARTDATE_Format **=** "%Y%m%d",

PRELIMINARY_TIME_SPAN **=** 365,

PRESCRIPTION **=** PRESCRIPTION,

PRESCRIPTION_ATC_COLNAME **=** "ATC",

PRESCRIPTION_ID_COLNAME **=** "ID",

PRESCRIPTION_DATE_COLNAME **=** "DATE",

ICD_INPUT **=** ICD_Entry,

DIAGNOSES **=** DIAGNOSES,

DIAGNOSES_ICD_COLNAME **=** "ICD",

DIAGNOSES_ID_COLNAME **=** "ID",

DIAGNOSES_DATE_COLNAME **=** "DATE",

INSURANTS **=** VERS,

INSURANTS_ID_COLNAME **=** "ID",

INSURANTS_BIRTH_YEAR_COLNAME **=** "DATEOFBIRTH",

MIN_INCLUSION_AGE **=** 18**)**

head(d)

ID DATEIndex STARTDATE DATEDIFF

1 1 2015**-**01**-**12 2011**-**01**-**01 1472 days

2 2 2012**-**08**-**05 2011**-**01**-**01 582 days

3 10 2012**-**01**-**12 2011**-**01**-**01 376 days

4 11 2013**-**11**-**10 2011**-**01**-**01 1044 days

5 13 2012**-**03**-**31 2011**-**01**-**01 455 days

6 14 2012**-**08**-**25 2011**-**01**-**01 602 days
