# Supplementary material for: A framework to build similarity-based cohorts for personalized treatment advice – a standardized, but flexible workflow with the R package SimBaCo
Source: PLoS One. 2020 May 29;15(5):e0233686. doi: 10.1371/journal.pone.0233686 (PMC7259608; doi:10.1371/journal.pone.0233686)
Supplement: S4 Appendix — (DOCX) [file pone.0233686.s008.docx]

**Appendix Part 4.** Search_IN_Dataframe() function call

Diag_ready **<-** Search_IN_Dataframe**(**DF1 **=** DIAGNOSES,

DF1_COLNAME **=** "ID",

DF2 **=** d,

DF2_COLNAME **=** "ID"**)**

nrow**(**Diag_ready**)** # From the diagnoses dataframe

**[**1**]** 48779

nrow**(**VO_ready**)** # From the prescription dataframe

**[**1**]** 64342

nrow**(**VERS_ready**)** # From the insurant dataframe

**[**1**]** 3513
