# Supplementary material for: A framework to build similarity-based cohorts for personalized treatment advice – a standardized, but flexible workflow with the R package SimBaCo
Source: PLoS One. 2020 May 29;15(5):e0233686. doi: 10.1371/journal.pone.0233686 (PMC7259608; doi:10.1371/journal.pone.0233686)
Supplement: S5 Appendix — (DOCX) [file pone.0233686.s009.docx]

**Appendix Part 5.** Draw_Scale_Chart() function call

Baseplot1 **<-** Draw_Scale_Chart**(**SELECT_COMORBIDITY **=** c**(**"hypunc","solidtum"**)**,

Change_Name_Plot **=** c**(**"Age","Hypertension\n uncomplicated","Solid tumor"**)**,

PATIENT_SIMILAR_BIRTH_YEAR **=** 1935,

PATIENT_SIMILAR_INDEXDATE **=** "20191210",

PATIENT_SIMILAR_INDEXDATE_FORMAT **=** "%Y%m%d",

PATIENT_SIMILAR_ICD= c**(**"I480","I100","C260"**)**,

PRESCRIPTION **=** VO_ready,

PRESCRIPTION_ATC_COLNAME **=** "ATC",

PRESCRIPTION_ID_COLNAME **=** "ID",

DIAGNOSES **=** Diag_ready,

DIAGNOSES_ICD_COLNAME **=** "ICD",

DIAGNOSES_ID_COLNAME **=** "ID",

DIAGNOSES_ICD_TYPE **=** "icd10",

INSURANTS **=** VERS_ready,

INSURANTS_ID_COLNAME **=** "ID",

INSURANTS_BIRTH_YEAR_COLNAME **=** "DATEOFBIRTH",

INSURANTS_INDEXDATE_COLNAME **=** "DATEIndex"**)**

Baseplot2 **<-** Draw_Scale_Chart**(**SELECT_COMORBIDITY **=** c**(**"diabunc","depre"**)**,

PATIENT_SIMILAR_BIRTH_YEAR **=** 1948,

Change_Name_Plot **=** c**(**"Age","ATC code

C09XXX","Prescriptions of ATC \n code C09XX",

"Depression","Diabetes\n uncomplicated"**)**,

PATIENT_SIMILAR_INDEXDATE **=** "20191210",

PATIENT_SIMILAR_INDEXDATE_FORMAT **=** "%Y%m%d",

PATIENT_SIMILAR_ICD**=** c**(**"I480","E119","F338"**)**,

PATIENT_SIMILAR_ATC **=** c**(**"^C09"**)**,

PATIENT_SIMILAR_ATC_COUNT **=** c**(**10**)**,

PRESCRIPTION **=** VO_ready,

PRESCRIPTION_ATC_COLNAME **=** "ATC",

PRESCRIPTION_ID_COLNAME **=** "ID",

DIAGNOSES **=** Diag_ready,

DIAGNOSES_ICD_COLNAME **=** "ICD",

DIAGNOSES_ID_COLNAME **=** "ID",

DIAGNOSES_ICD_TYPE **=** "icd10",

INSURANTS **=** VERS_ready,

INSURANTS_ID_COLNAME **=** "ID",

INSURANTS_BIRTH_YEAR_COLNAME **=** "DATEOFBIRTH",

INSURANTS_INDEXDATE_COLNAME **=** "DATEIndex"**)**
