# Supplementary material for: A framework to build similarity-based cohorts for personalized treatment advice – a standardized, but flexible workflow with the R package SimBaCo
Source: PLoS One. 2020 May 29;15(5):e0233686. doi: 10.1371/journal.pone.0233686 (PMC7259608; doi:10.1371/journal.pone.0233686)
Supplement: S6 Appendix — (DOCX) [file pone.0233686.s010.docx]

**Appendix Part 6.** Find_Similar() function call

Nearest_pat_1 **<-** Find_Similar(PATIENT_SELECTION = "NEW",

ANALYSIS_TYPE = "NEAREST",

DISTANCE_MEASURE = "GOWER",

SELECT_COMORBIDITY = c("hypunc","solidtum"),

PERCENT_CUT_OFF = 10,

PATIENT_SIMILAR_BIRTH_YEAR = 1935,

PATIENT_SIMILAR_INDEXDATE = "20191030",

PATIENT_SIMILAR_INDEXDATE_FORMAT = "%Y%m%d",

PATIENT_SIMILAR_ICD= c("I480","I100","C260"),

PRESCRIPTION = VO_ready,

PRESCRIPTION_ID_COLNAME = "ID",

DIAGNOSES = Diag_ready,

DIAGNOSES_ICD_COLNAME = "ICD",

DIAGNOSES_ID_COLNAME = "ID",

DIAGNOSES_ICD_TYPE = "icd10",

INSURANTS = VERS_ready,

INSURANTS_ID_COLNAME = "ID",

INSURANTS_BIRTH_YEAR_COLNAME = "DATEOFBIRTH",

INSURANTS_INDEXDATE_COLNAME = "DATEIndex")

Nearest_pat_2 **<-** Find_Similar(PATIENT_SELECTION = "NEW",

ANALYSIS_TYPE = "NEAREST",

DISTANCE_MEASURE = "HEOM",

SELECT_COMORBIDITY = c("diabunc","depre"),

PERCENT_CUT_OFF = 10,

PATIENT_SIMILAR_BIRTH_YEAR = 1948,

PATIENT_SIMILAR_INDEXDATE = "20191030",

PATIENT_SIMILAR_INDEXDATE_FORMAT = "%Y%m%d",

PATIENT_SIMILAR_ATC = c("^C09"),

PATIENT_SIMILAR_ATC_COUNT = c(10),

PATIENT_SIMILAR_ICD= c("I480","E119","F338"),

PRESCRIPTION = VO_ready,

PRESCRIPTION_ATC_COLNAME = "ATC",

PRESCRIPTION_ID_COLNAME = "ID",

DIAGNOSES = Diag_ready,

DIAGNOSES_ICD_COLNAME = "ICD",

DIAGNOSES_ID_COLNAME = "ID",

DIAGNOSES_ICD_TYPE = "icd10",

INSURANTS = VERS_ready,

INSURANTS_ID_COLNAME = "ID",

INSURANTS_BIRTH_YEAR_COLNAME = "DATEOFBIRTH",

INSURANTS_INDEXDATE_COLNAME = "DATEIndex")

nrow**(**Nearest_pat_1**)**

**[**1**]** 351

nrow**(**Nearest_pat_2**)**

**[**1**]** 351
