# Supplementary material for: A framework to build similarity-based cohorts for personalized treatment advice – a standardized, but flexible workflow with the R package SimBaCo
Source: PLoS One. 2020 May 29;15(5):e0233686. doi: 10.1371/journal.pone.0233686 (PMC7259608; doi:10.1371/journal.pone.0233686)
Supplement: S8 Appendix — (DOCX) [file pone.0233686.s012.docx]

**Appendix Part 7.** Kaplan Meier Analysis of the cohorts

First, we subdivide our data source again according to the patients (IDs) included in the precision cohorts:

VO_ready1 **<-** Search_After_Index**(**DF1 **=** VO_ready,

DF1_COLNAME **=** "ID",

DF1_DATENAME **=** "DATE",

DF1_CODE **=** "ATC",

DF2 **=** Index_ready,

DF2_COLNAME **=** "ID",

DF2_DATENAME **=** "DATEIndex"**)**

VERS_ready1 **<-** Search_After_Index**(**DF1 **=** VERS_ready,

DF1_COLNAME **=** "ID",

DF1_DATENAME **=** "DATEOFDEATH",

DF1_CODE **=** "SEX",

DF2 **=** Index_ready,

DF2_COLNAME **=** "ID",

DF2_DATENAME **=** "DATEIndex"**)**

nrow**(**VO_ready1**)**

**[**1**]** 5285

nrow**(**VERS_ready1**)**

**[**1**]** 351

Second, we introduce a summary function putting the data into the right format for a survival analysis and drawing Kaplan-Meier plots based on the survival and survminer R packages:

Kaplan_Data **<-** Data_Plot_Similarity**(**ATC_INPUT **=** c**(**"B01AF01","B01AE07"**)**,

OUTCOME **=** "DEATH",

PRESCRIPTION **=** VO_ready1,

PRESCRIPTION_ID_COLNAME **=** "ID",

PRESCRIPTION_CODE_COLNAME **=** "CODE",

PRESCRIPTION_CODE_DATUM_COLNAME **=** "CODE_DATUM",

PRESCRIPTION_INDEXDATUM_COLNAME **=** "INDEXDATUM",

DIAGNOSES **=** Diag_ready1,

DIAGNOSES_ID_COLNAME **=** "ID",

DIAGNOSES_CODE_COLNAME **=** "CODE",

DIAGNOSES_CODE_DATUM_COLNAME **=** "CODE_DATUM",

DIAGNOSES_INDEXDATUM_COLNAME **=** "INDEXDATUM",

INSURANTS **=** VERS_ready1,

INSURANTS_ID_COLNAME **=** "ID",

INSURANTS_CODE_COLNAME **=** "CODE",

INSURANTS_CODE_DATUM_COLNAME **=** "CODE_DATUM",

INSURANTS_INDEXDATUM_COLNAME **=** "INDEXDATUM"**)**

Further explanations for the parameters are listed in **S4 Table**. After running these code lines for exemplary case one, we obtain the following data frame:

head**(**Kaplan_Data**)**

ID TIME OUTCOME CODE

2 273 1 B01AE07

2 117 0 B01AE07

13 51 1 B01AE07

13 1 0 B01AE07

19 92 1 B01AE07

22 618 1 B01AE07

nrow**(**Kaplan_Data**)**

**[**1**]** 868

After creating this data frame, we use the survival and survminer packages as described below, to draw the Kaplan-Meier-Plots.

library**(**survival**)**

library**(**survminer**)**

library**(**dplyr**)**

df **<-** Kaplan_Data

df **<-** df %>%

filter**(**df**$**OUTCOME **==** 1**)**

surv_object **<-** Surv**(**time **=** df**$**TIME, event **=** df**$**OUTCOME**)**

fit1 **<-** survfit**(**surv_object **~** df**$**CODE, data **=** df**)**

plot **<-**ggsurvplot**(**fit1, data **=** df, risk.table **=** **FALSE**, xlim **=** c**(**0,750**)**, break.time.by**=**365, xlab **=** "Time in days",palette **=** c**(**"#003366", "#CC6666"**)**, legend **=** "none", ylab**=**"",

ggtheme**=**theme_survminer**(**font.legend**=**c**(**12,"bold","black"**)))**

**+** guides**(**colour **=** guide_legend**(**nrow **=** 2**))**
